# Wetting Behavior of Kerogen Surfaces: Insights from Molecular Dynamics

**DOI:** 10.1021/acs.langmuir.3c03367

**Published:** 2024-03-07

**Authors:** Neda Sanchouli, Saeed Babaei, Matej Kanduč, Fatemeh Molaei, Mehdi Ostadhassan

**Affiliations:** † Department of Petroleum Engineering, Shahid Bahonar University of Kerman, Kerman 7616914111, Iran; ‡ Civil Engineering Faculty, K. N. Toosi University of Technology, Tehran 1969764499, Iran; § Department of Theoretical Physics, Jožef Stefan Institute, Jamova 39, Ljubljana 1000, Slovenia; ∥ Department of Mining and Geological Engineering, The University of Arizona, Tucson, Arizona 85721, United States; ⊥ Stantec consulting company, Ann Arbor, Michigan 48108, United States; # Institute of Geosciences, Marine and Land Geomechanics and Geotectonics, Christian-Albrechts Universität, Kiel 24118, Germany

## Abstract

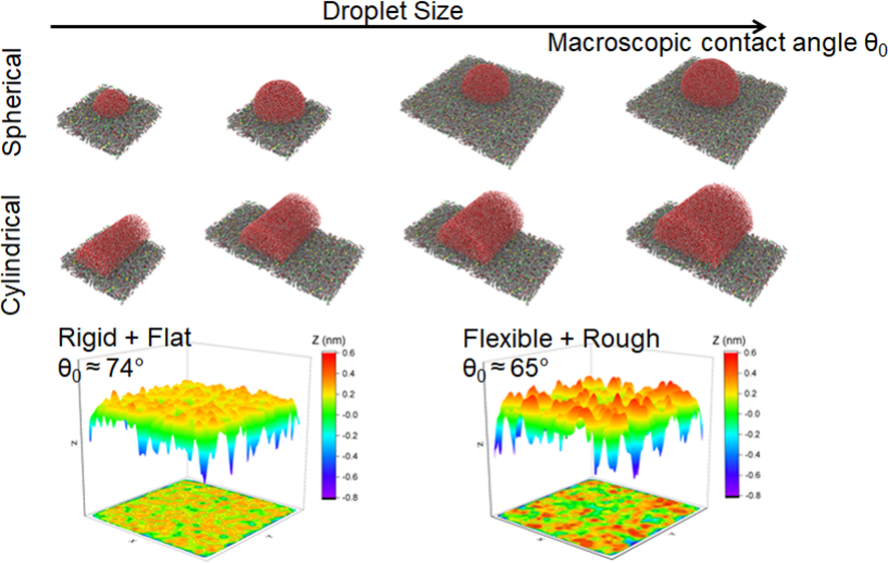

In this study, the wettability of a kerogen surface, a key component of shale reservoirs, is investigated by using molecular dynamics simulations. Specifically, we examined the impact of droplet size and morphology as well as surface roughness on the water contact angles. The findings highlighted that the contact angle dependency on the droplet size intensifies with increased rigidity of the surface. Conversely, as the surface becomes more flexible and rougher, it gains hydrophilicity. The higher hydrophilicity stems from the ability of water molecules to penetrate the kerogen corrugations and form more hydrogen bonds with heteroatoms, particularly oxygen. Notably, the contact angle of kerogen hovers between 65 and 75°, thereby crossing the transition from an underoil hydrophilic to an underoil hydrophobic state. Consequently, minor alterations in the kerogen nanostructure can dramatically alter the wetting preference between water and oil. This insight is of paramount significance for refining strategies in managing fluid interactions in shale reservoirs such as geological carbon storage or oil extraction.

## 1 Introduction

The extraction of hydrocarbons from shale formations has revolutionized the energy industry owing to the advancements in hydraulic fracturing and horizontal drilling techniques.^1^ In this respect, hydraulic fracturing involves injecting pressurized gas or water into the reservoir, creating fractures in the rock matrix to establish the necessary pathways for reservoir fluids to flow. Herein, poor recovery of the hydraulic fracturing flow back water is one of the challenges that has been widely observed in field operations.^2^ This issue is generally related to the distribution of fluids in porous media, which makes the wettability play a crucial role, ultimately impacting production rates and the effectiveness of the entire operations. It is important to note that shale has an ultralow permeability and complex mineral compositions composed of clay and other minerals as well as organic matter. In addition to the permeability, organic-rich and liquid-rich shale strata are known to have a complex pore network with a wide range of pore sizes from micro- to macropores (based on IUPAC pore size classification).^3,4^ The wettability behavior of kerogen is highly intricate. As kerogen matures, there is a corresponding decrease in the oxygen-to-carbon (O/C) ratio,^5,6^ leading to a decrease in hydrophilicity and an increase in the contact angle. In contrast, increased kerogen maturity correlates with greater surface roughness,^7^ which can affect the contact angle. All in all, this makes the determination of wettability in shales very challenging and scale-dependent.^8^

Kerogen is an essential part of the organic matter not soluble in common polar solvents.^5,9^ The general consensus has been that kerogen is mainly hydrophobic;^2^ however, recent studies have observed that the presence of heteroatoms (polar nitrogen-, sulfur-, and oxygen-containing (NSO) compounds) in kerogen might induce a mixed-wet or even hydrophilic characteristics in kerogen, leading to an intricate wetting behavior of this matter.^10−12^ Thus, obtaining an accurate knowledge of kerogen wettability and gaining better insight into how kerogen impacts fluid flow can profoundly help not only in production from unconventional shale but also in effective geological carbon sequestration (GCS) since shale reservoirs have shown promising results to act as CO_2_ sinks. CO_2_ can be stored in shale reservoirs through the residual or capillary trapping mechanism, by which carbon is trapped through high capillary forces in the porous media of the formation rock.^13,14^

To bridge our understanding of the intricacies of wetting phenomena with the molecular-level nuances, molecular dynamics (MD) simulations have emerged as an indispensable tool.^15−17^ Most MD simulations, nevertheless, have primarily focused on homogeneous and smooth surfaces such as graphene and graphite,^18,19^ but these do not properly represent kerogen’s heterogeneous and rough nature.^20,21^ To gain deeper insights into wettability, recent studies have started to employ more realistic models of kerogen in which wettability is described by the wetting contact angle of the fluid on the kerogen surface (Table 1).

**Table 1:** Summary of MD Studies on Contact Angles of Different Types of Kerogen Surfaces

	kerogen surface							
ref.	type	surface state	dimension (Å^3^)	effect of roughness	number of H_2_O molecules	droplet shape	effect of droplet shape	effect of droplet size
Ho et al.^10^	II-D	flexible	89.67 × 103.66 × 100	-	1100	spherical	-	-
Li et al.^11^	II-D	rigid	112 × 61.4 × 140	-	1500	cylindrical	-	-
Ho and Wang^13^	II-D	flexible	89.67 × 103.66 × 100	-	1100	spherical	-	-
Zhou et al.^14^	II-D	rigid	110 × 135 × 36.5	-	8000	cylindrical	-	-
Yang et al.^22^	II-D	flexible	194.86 × 34.25 × 21.12	-	2000	cylindrical	-	-
Zhou et al.^23^	II-C	N/A	154.6 × 80.5 × 20	-	N/A	cylindrical	-	-
Jagadisan and Heidari^24^	I-A	rigid	N/A	-	1728	spherical	-	-
	II-A							
	II–B							
	II-C							
	II-D							
	III-C							

**Figure 1:**
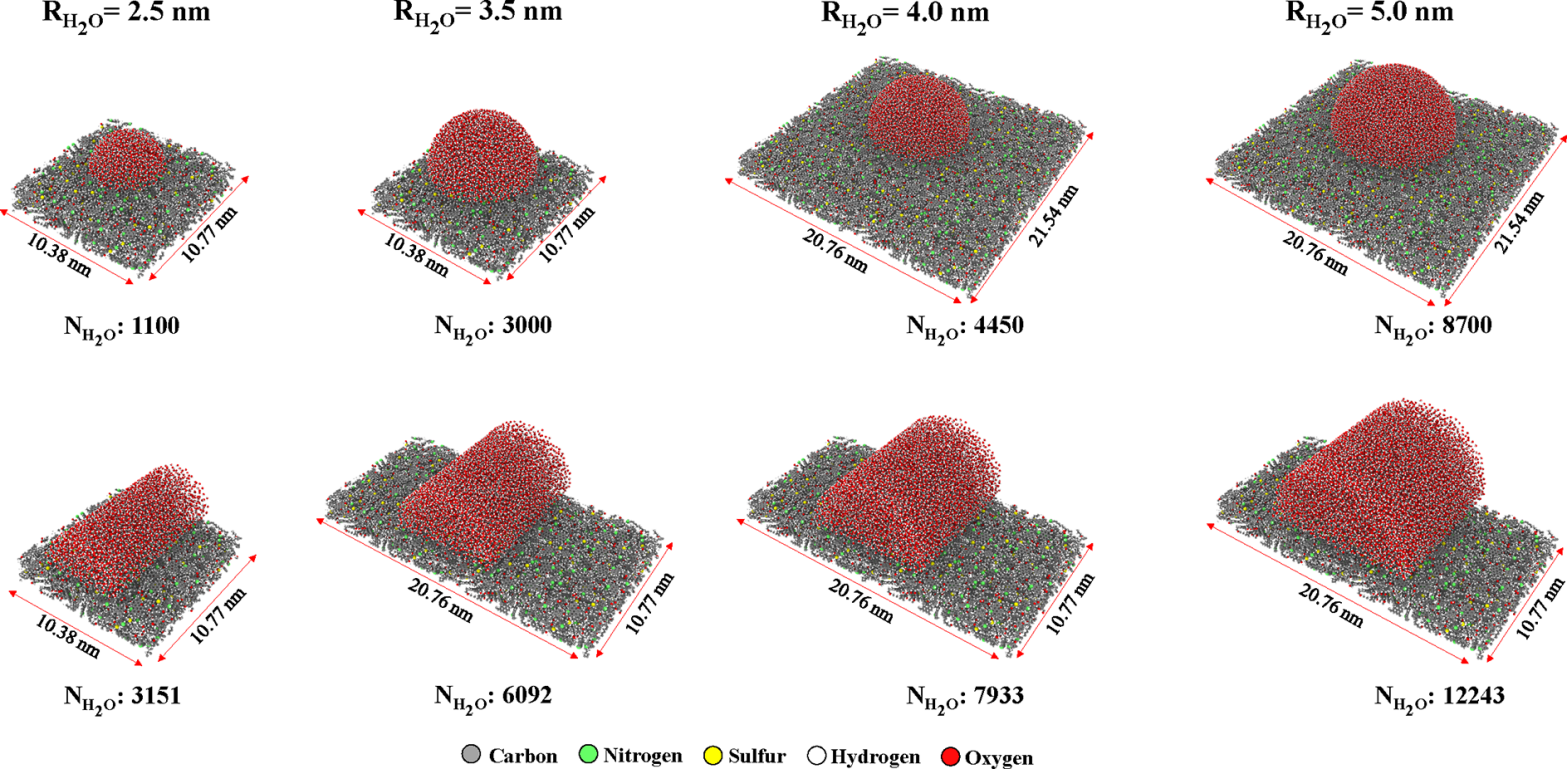
Initial configurations of spherical and cylindrical droplets on the kerogen surface.

Unlike experimental studies, employing a macroscopic-scale droplet in MD simulations is unfeasible due to the considerable computational demands. Consequently, nanodroplets have been widely used in such simulations. However, nanodroplets exhibit certain differences compared to macroscopically large droplets, as used in experiments. In fact, the contact angle of very small droplets depends on the droplet size,^25−27^ as described by the modified Young’s equation:^28^
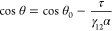
1where θ is the contact angle of the (nano)droplet, θ_0_ is Young’s contact angle of a macroscopically large droplet at equilibrium, α is the droplet’s radius, γ_12_ is the interfacial tension of the fluids, and τ is the apparent line tension. The original concept of line tension, as introduced by Gibbs, refers to the excess free energy per unit length of the three-phase contact line of the droplet. However, in wetting processes, other factors also contribute to cos θ, which scale inversely with the radius and thus mask the “actual” three-phase contact line tension. Some of these contributing physical effects do not even originate from the three-phase contact line. These factors include the line tension variation with instantaneous contact angle (line tension stiffness), the curvature-dependent surface tension (Tolman correction), and the effect of the substrate dimpling beneath the droplet.^29,30^ Consequently, the apparent line tension, τ, is an empirical parameter consolidating all size-dependent factors affecting the contact angle.^25,31^ In this regard, the apparent line tension heavily depends on the type of fluids, surface roughness, and chemical heterogeneities, with reported values ranging from 10^–12^ to 10^–5^ N and of either sign.^32,33^ Furthermore, there are conflicting findings regarding how the droplet’s morphology, being cylindrical or spherical, influences the apparent line tension. In the case of cylindrical droplets, the use of periodic boundary conditions leads to the realization of an infinitely long configuration (Figure 1). Some studies have suggested that using a cylindrical droplet could reduce scale effects by eliminating the impact of three-phase contact line tension, thus leading to contact angles close to macroscopic ones.^15,34^ However, it has been demonstrated that despite eliminating the effect of the three-phase contact line by its design, the apparent line tension stemming from other effects, as mentioned above, may persist and affect the contact angle of cylindrical droplets at the nanoscale.^35^ Consequently, there is no general consensus about the effect of the droplet’s shape on the nanoscale contact angle.

One of the factors affecting the macroscopic contact angle and apparent line tension is surface roughness. Given that the kerogen structure is neither smooth nor crystalline, modeling it with varying degrees of roughness can provide valuable insights into how such surface textures influence the apparent line tension and contact angle. The most pragmatic approach to achieve this involves modulating the surface “flexibility”, which relates to the ability of surface atoms for movement and displacement, resulting in nanoscopic roughness. As summarized in Table 1, while several studies have explored both rigid and flexible states of the kerogen surface, the specific impact of surface roughness on the contact angle remains an unexplored aspect. To the best of our knowledge, previous studies on kerogen surfaces have primarily focused on the qualitative evaluation of alterations in the water contact angle in the presence of varying CO_2_ pressures, indicating that increasing CO_2_ pressure leads to an increasing water contact angle.^13,14^ However, an accurate assessment of the macroscopic water contact angle using varying droplet sizes has not been adequately conducted to date. Furthermore, the influence of kerogen roughness (modeled via varying the flexibility) on the contact angle is not yet fully understood. Therefore, in this study, we employed MD simulations to assess the macroscopic water contact angle on type II-C kerogen surfaces in both rigid and flexible states. These kerogen types were chosen as models due to their significant potential for gas production. We used spherical and cylindrical droplets of four different radii and applied the modified Young’s equation (eq 1) to determine the macroscopic contact angles for the two surfaces. The line tensions for both configurations on rigid and flexible surfaces are also investigated. Additionally, we quantified the roughness of the surface using two methods, followed by an analysis of the number of hydrogen bonds for rigid and flexible kerogen.

## 2 Materials and Methods

### 2.1 Simulation Details

MD simulations were conducted using the LAMMPS package.^36^ Among the three types of kerogen, type II is commonly found in marine environments and has relatively high H/C and low O/C ratios.^9^ In this work, we studied the wettability of type II-C kerogen (Figure S1 and Table S1 in the Supporting Information (SI)) using the realistic model presented by Ungerer et al.,^5^ which is oil-prone and abundant in organic shale. We used 50 kerogen macromolecules of type II-C with a chemical formula of C_242_H_219_O_13_N_5_S_2_ to construct the kerogen matrix. Further information on the kerogen surface construction method can be found in our previous work.^37^ The constructed kerogen slab measured 10.38 × 10.77 × 2.39 nm^3^ in size, and it was replicated in some cases to cover the entire simulation box (see further in the text). The density of the kerogen matrix is 1.19 g/cm^3^, which falls within the range of experimental values for type II kerogen of 1.18–1.35 g/cm^3^.^38^ The consistent valence force field was utilized for the kerogen macromolecules.^39^

Figure 1 provides a summary of the initial droplet radii, the number of H_2_O molecules, and the dimensions of the kerogen surfaces in the simulation systems. Spherical and cylindrical water droplets of four different sizes were placed on the kerogen surface to assess the influence of the droplet’s shape and size on the contact angle. To prevent droplets from interacting with their periodic images while also minimizing computational costs, the lateral dimensions of the simulation box were adjusted based on the droplet size, specifically 10.38 × 10.77 nm^2^ (for spherical droplet radii of 2.5 and 3.5 nm and cylindrical droplet radii of 2.5 nm), 20.76 × 10.77 nm^2^ (for cylindrical droplet radii of 3.5, 4.0, and 5.0 nm), and 20.76 × 21.54 nm^2^ (for spherical droplet radii of 4.0 and 5.0 nm). Furthermore, to gain a comprehensive understanding of how the kerogen nanostructure affects wettability, we investigated two distinct conditions on the kerogen surface. The first condition involved a *rigid* state, where the kerogen atoms were “frozen” in place, with their velocities held at zero. This rigid state represents an atomistically flat, static, and unchanging surface. In contrast, the *flexible* state allowed the kerogen atoms to move freely according to the laws of motion. Because of these atomic movements, this surface is rougher and can adapt to external conditions.

Contact angle simulations were performed in an NVT ensemble at 300 K. The temperature was controlled using the Nosé–Hoover thermostat^40,41^ with a relaxation time of 0.1 ps. Water molecules were simulated using the SPC/E model,^42^ with the SHAKE algorithm employed to constrain bond stretching and angle bending within these molecules.^43^ The SPC/E model was used because it has demonstrated good results for the structure and dynamic properties of liquid water.^44^ The pairwise Coulomb and Lennard-Jones (LJ) potentials represented the nonbonded interactions. A cutoff distance of 1.2 nm was used to compute the LJ and the short-range part of the electrostatic interactions. Recent research^45−52^ has indicated that tail correction can reduce the sensitivity to the chosen cutoff distance in the interfacial properties. Therefore, this study implemented tail corrections to estimate the LJ energy beyond the cutoff region. The long-range electrostatic interactions were computed using the particle–particle particle–mesh solver with an accuracy of 10^–4^. Lorentz–Berthelot mixing rules^53^ were applied to calculate interactions between different atom types with periodic boundary conditions in all directions.

The total simulation run for droplets with initial radii of 2.5 and 3.5 nm was 10 ns, and for those with initial radii of 4.0 and 5.0 nm, it was 15 ns. Equations of motion were integrated using the velocity Verlet algorithm^54^ with a time step of 1 fs. In all simulations, the first 5 ns was allocated for reaching equilibrium, with the remaining simulation time dedicated to calculating the contact angle. During this production phase, atom positions were stored every 5 ps. Finally, four independent MD simulations for each droplet size were conducted, each initiated with different initial configurations. The average contact angle and standard deviation for each droplet size were evaluated by averaging the results from the four independent trajectories.

### 2.2 Contact Angle Calculation

**Figure 2:**
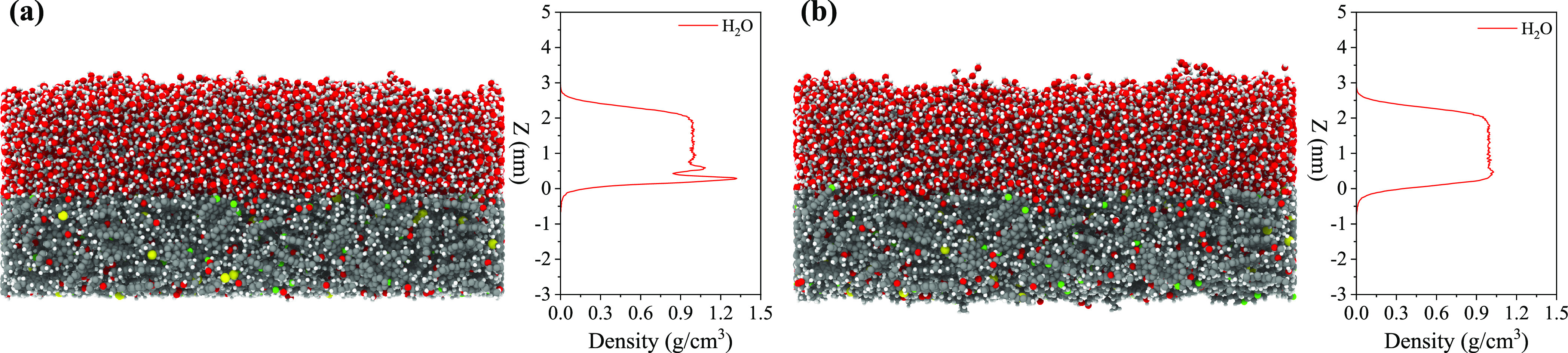
Final configurations and density profiles of water films on the kerogen surface, featuring (a) the rigid and (b) flexible state.

To calculate the contact angle of a droplet, it is necessary to define the effective position of the water–surface interface. The best practice is to identify this as the Gibbs dividing surface (GDS) of the water phase. For simplicity in numerical analysis, we determined the GDS by depositing a 3 nm-thick water film on the kerogen surface with dimensions of 10.37 × 10.76 nm^2^. This approach circumvents the complexities typically encountered with droplet-based approaches such as substrate deformations beneath the droplet. Simulations lasting 2 ns were conducted for both rigid and flexible cases in the NVT ensemble. Figure 2 shows the final configuration snapshots and water density profiles in the *z*-direction.^55^

**Figure 3:**
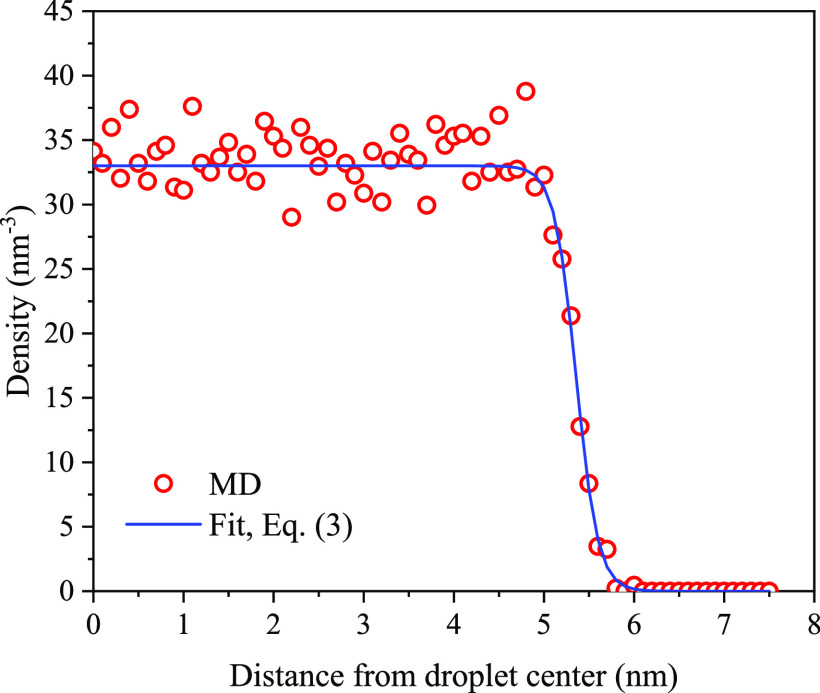
Water density profile along the *x*-direction in the first layer of the 5 nm cylindrical water droplet on the rigid kerogen surface. The solid line is the fit of eq 3 to the MD data points.

The position of the GDS was calculated by integrating over the water density profile ρ(*z*):
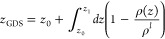
2

where *z*_0_ is a position outside the water slab, where its density is virtually zero, and *z*_1_ is the position inside the water slab, where the density reaches the bulk value ρ^*l*^ = 33 nm^–3^.^55^ To identify the water–vapor interface of a cylindrical droplet, we calculated the center of mass (COM) of the droplet for each snapshot as it moved throughout the simulation. This COM serves as the origin of the *x*-axis. We then discretized the *z*-axis into 0.2 nm bins. Subsequently, we calculated the water density in each bin along the *x*-axis, distinguishing between the left (*x* < 0) and the right (*x* > 0) side (Figure 3). By employing the density profile and following the procedure described by de Ruijter et al.,^56^ the liquid–gas interface of the water droplet was identified by fitting the profile with a hyperbolic tangent function:
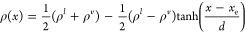
3where ρ^*l*^ = 33 nm^–3^ as in eq 2, ρ^*v*^ is considered to be 0, *x*_e_ represents the location where the water density is halfway between its liquid bulk and vapor phase value, and *d* represents the width of the water–vapor interface. The sigmoidal fit provided *x*_e_ as the boundary of the water–vapor interface within each *z*-bin. Subsequently, a circle was fitted to the interface positions. The point where the water-kerogen GDS intersected with this fitted circle was utilized to calculate the contact angle of the droplet. The same method was applied to spherical droplets for calculating the water–vapor interface, but the density in the *x*-direction, ρ(*x*), was replaced by the radial density, ρ(*r*).

## 3 Results and Discussion

### 3.1 Contact Angles

**Figure 4:**
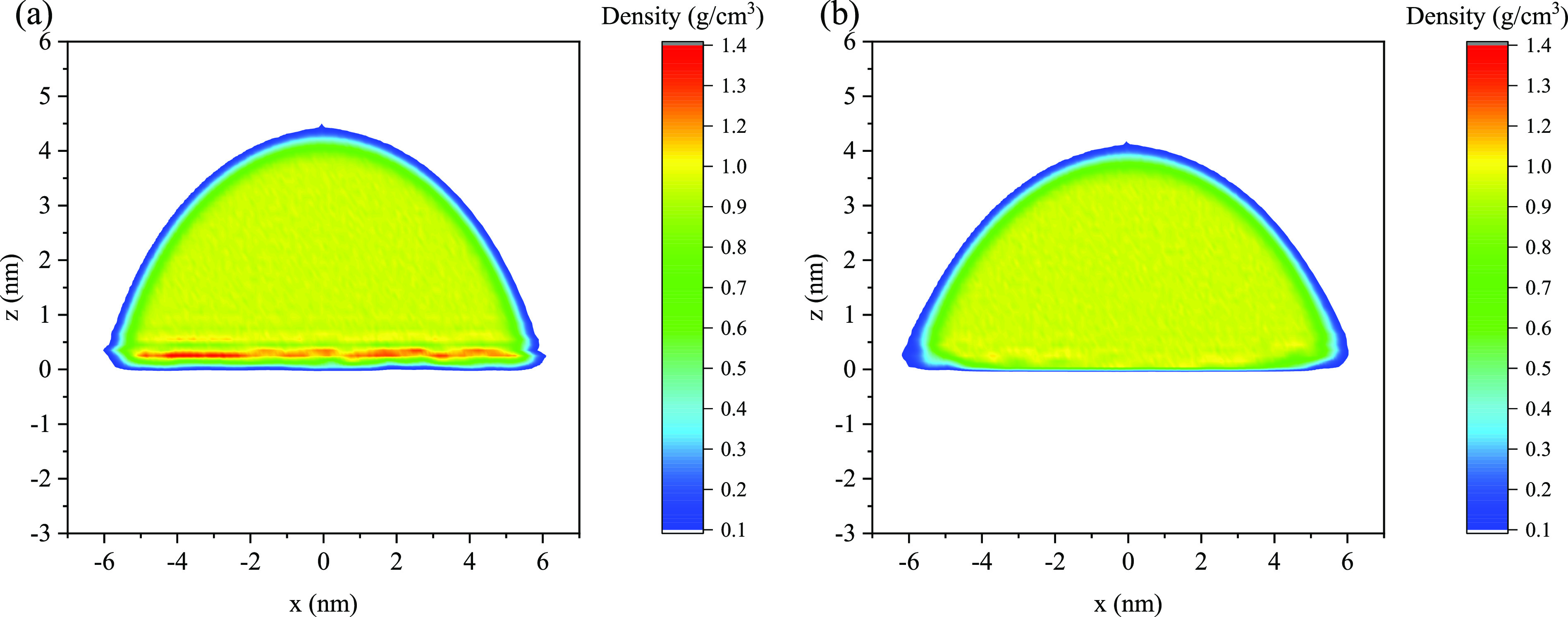
Density plot of an equilibrated cylindrical water droplet on the (a) rigid and (b) flexible kerogen surface.

We begin with Figure 4, which shows the density plots of a cylindrical water droplet after equilibration on both rigid and flexible surfaces. Corresponding plots for a spherical droplet are available in the SI (Figure S2). On the flexible substrate, a slight deformation of the droplet’s base near the contact line may be noticeable, where surface tension pulls the substrate upward, creating a “wetting ridge”. This upward pull is counteracted by Laplace pressure, inducing a dimpled effect beneath the droplet. The height of this wetting ridge is on the order of the elastocapillary length, which is the ratio of surface tension to the Young’s modulus of the surface.^30,57,58^ The associated elastic energy is proportional to the circumference of the three-phase contact line, thereby contributing to line tension but not affecting the macroscopic contact angle.^30^ This elastic deformation also alters the local contact angle.^59^ However, our study focuses on the apparent (global) contact angle, measured relative to the plane of the substrate.

Another key observation from Figure 4 is the tendency of water molecules to form distinct layers on the stiff substrate, as observed also for the water film in Figure 2a. Such layering is a typical occurrence at solid flat surfaces.^60^ The layering diminishes within a span of a few water molecules. The extent of this pattern enables us to estimate the impact of the kerogen surface on the water behavior, particularly in terms of disjoining pressure. Beyond this structured zone, the properties of water become bulk-like, which constitutes the majority of a droplet in our simulations.

**Figure 5:**
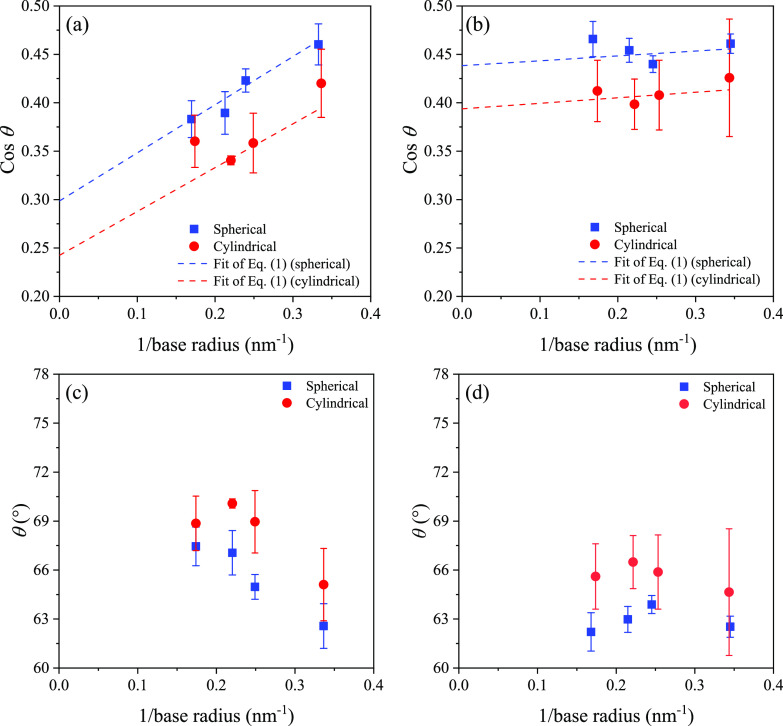
Cosine of the measured contact angles versus inverse base radii on (a) rigid and (b) flexible kerogen surfaces. The dashed lines are fits of eq 1 to the contact angle data. Contact angles of spherical and cylindrical droplets versus the inverse base radius on (c) rigid and (d) flexible kerogen surfaces. The error bars represent the uncertainties based on four independent simulations for each droplet size.

The contact angles evaluated for both spherical and cylindrical droplets on the surfaces of rigid and flexible kerogen structures are plotted against the inverse base radii in Figure 5a,b. On the rigid surface, contact angles show a notable increase with droplet size, whereas on the flexible surface, there is insignificant growth in contact angles as the droplet size increases. This distinction appears to be rooted in subtle differences between the two surface types, particularly in terms of flexibility and roughness. On the flexible surface, water molecules are able to penetrate the surface corrugations. Through visual examination of the simulation trajectories, we noted that droplets are more laterally mobile on the rigid kerogen surface than on the flexible kerogen surface, implying higher friction on the latter. The droplet dynamics are, however, beyond the scope of this study. Additionally, both cylindrical and spherical droplets demonstrate similar variations in contact angle with size on both types of kerogen surfaces. This observation contrasts some previous studies that suggested that cylindrical droplets are less influenced by their size.^33^

By fitting eq 1 to the contact angle data on the rigid surface in Figure 5a, we obtain the macroscopic contact angle, corresponding to the extrapolation 1/base radius = 0. To account for uncertainties, instead of using the least-squares of absolute values, we use the least-squares of values normalized by their corresponding error bars.^61^ For spherical droplets on the rigid surface, we obtain θ_0_ = 72.6 ± 1.5°, while for cylindrical droplets, θ_0_ = 76 ± 4° (Figure 5a). In the same way, we calculated the macroscopic contact angles on the flexible surface as 64 ± 2° for the spherical droplet and 67 ± 2° for the cylindrical droplet (Figure 5b). When extrapolated to the macroscopic contact angle, both droplet morphologies yield identical results within the bounds of numerical uncertainty, which is consistent with our expectations. For better visualization, we also plot the contact angles θ as a function of the inverse base radii in Figure 5c,d.

The results indicate that factors such as surface flexibility and roughness are crucial in governing the contact angle. As the kerogen surface gains roughness, its hydrophilic character is slightly intensified, resulting in a lower water contact angle. This differentiation also leads to different apparent line tensions of the two morphologies, which we analyze in the following.

**Figure 6:**
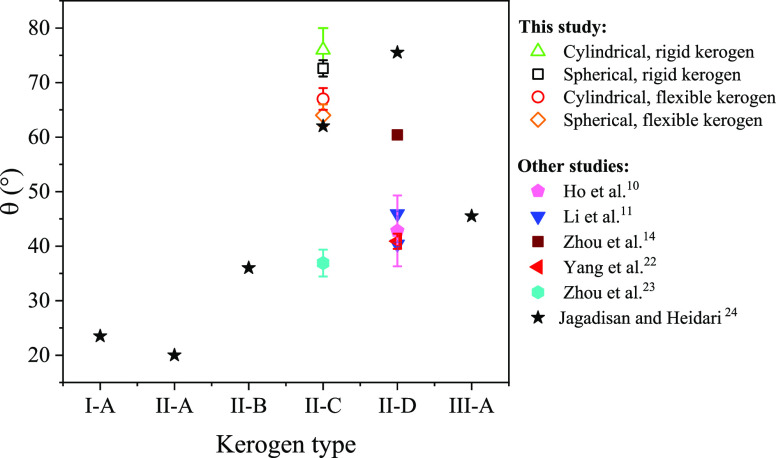
Comparison of the macroscopic contact angles calculated in this study with the results of other MD simulations^10,11,14,22−24^ across various kerogen types.

In Figure 6, we present the macroscopic water contact angles obtained from our simulations alongside data from other MD simulation studies^10,11,14,22−24^ on various kerogen surfaces. It is evident that the contact angles found in our study fall within the established range. The noticeable variations in contact angle values can be ascribed to multiple factors, including kerogen type, droplet shape, and surface roughness.

### 3.2 Line Tension

From the fits to the MD data in Figure 5a,b, we also extract the apparent line tensions. Using γ = 63.6 mN/m (for the SPC/E water model at 300 K^62^), the calculated apparent line tensions for spherical and cylindrical droplets on the rigid surface are −32 ± 6 and −29 ± 19 pN, respectively. Because of the limits of numerical accuracy, resolving the difference in the apparent line tensions between the two droplet morphologies is not possible. We can only conclude that the actual three-phase contact line tension is smaller than the numerical accuracy achieved in this study.

On the flexible surface, the apparent line tensions for spherical and cylindrical droplets are −3 ± 7 and −4 ± 8 pN, respectively. In other words, they are effectively nonexistent within the numerical accuracy, which is ∼10 pN in this case. Similar to the case for rigid surface, discerning the three-phase contact line tension from the difference between the two apparent line tensions is not possible. Nevertheless, the obtained results align with previous studies, which suggest that line tensions are typically on the order of tens of pN.^31^

### 3.3 Roughness

To understand the differing contact angles observed between the rigid and flexible surfaces, it is important to consider the role of surface roughness. Young’s equation is commonly used to calculate the contact angle of a droplet on a smooth and homogeneous surface. However, in reality, surfaces are often rough, which impacts the liquid–surface interactions and consequently the contact angle. When a surface deviates from the ideal smooth state, Young’s equation may no longer accurately describe the contact angle. In such scenarios, the Wenzel equation offers a more accurate description. The Wenzel equation relates the contact angle (θ_Rough_) observed on the rough surface to the contact angle (θ_Smooth_) that would be observed on an ideal, perfectly smooth version of the same surface, via the relation:^63^
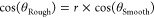
4Here, the coefficient *r* > 1 stands for the Wenzel roughness and takes into account the amplification effect of surface roughness on the contact angle, effectively accounting for the increased contact area between the liquid and rough surface. This wetting model assumes that the droplet infiltrates the corrugations of the rough surface. For a hydrophilic substrate (θ_Smooth_ < 90°), increasing the roughness enhances the surface’s wettability. Conversely, for a hydrophobic substrate (θ_Smooth_ > 90°), the contact angle increases with the roughness and the droplet may not even infiltrate the corrugations.

Within our simulation model, the rigid kerogen surface can be effectively considered nearly ideally smooth. Consequently, the macroscopic contact angle observed on the rigid surface can be regarded as θ_Smooth_. Conversely, the flexible surface is treated as inherently rough, yielding a corresponding contact angle of θ_Rough_. By applying eq 4, we compute the Wenzel roughness values obtained from the analysis of both spherical and cylindrical droplets, which yield 1.5 ± 0.2 and 1.6 ± 0.5, respectively (see Table 2). The results are indistinguishable within the given uncertainty.

**Table 2:** Roughness of the Flexible Kerogen Surface Using the Wenzel Model and the SASA Method, along with the Ratio of Surface–Water Hydrogen Bonds between the Flexible and Rigid Kerogen (HB Ratio)

droplet shape	Wenzel roughness	SASA-based roughness	HB ratio
spherical	1.5 ± 0.2	1.53 ± 0.06	1.11 ± 0.04
cylindrical	1.6 ± 0.5		

**Figure 7:**
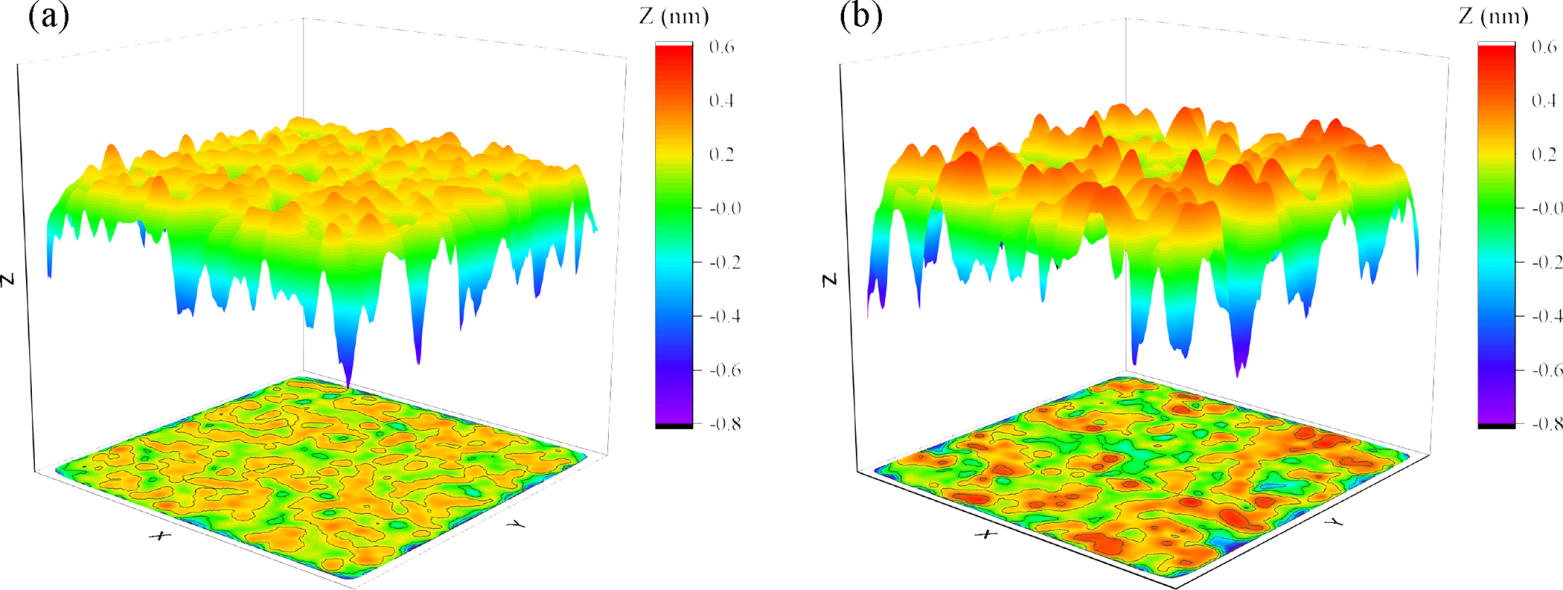
Surface profiles of (a) rigid and (b) flexible kerogen surfaces.

The roughness of a surface can also be estimated by using a direct approach that involves calculating the surface area of rigid and flexible kerogen. Here, we used the Visual Molecular Dynamics (VMD) tool^64^ to calculate each kerogen slab’s solvent-accessible surface area (SASA). This was done by considering a water molecule as a probe particle with a radius of 0.14 nm (approximately the size of a water molecule), allowing us to record the locations of the probe particles on the surfaces (Figure 7). Then, the surface roughness was estimated from these data following eq 5:
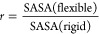
5

To that end, we carried out 2 ns simulations of a water film on both rigid and flexible kerogen surfaces. For the rigid surface, where the kerogen atoms were “frozen”, a single snapshot was used to compute the surface area. In the case of the flexible state, we determined the surface area from 20 distinct snapshots. Based on eq 5, the SASA roughness was calculated to be 1.53 (see Table 2). Thus, the SASA-based prediction aligns closely with both values for Wenzel roughness. Hence, the reduced contact angles observed on flexible surfaces can be attributed to their roughness and increased effective area.

### 3.4 Hydrogen Bonds

**Figure 8:**
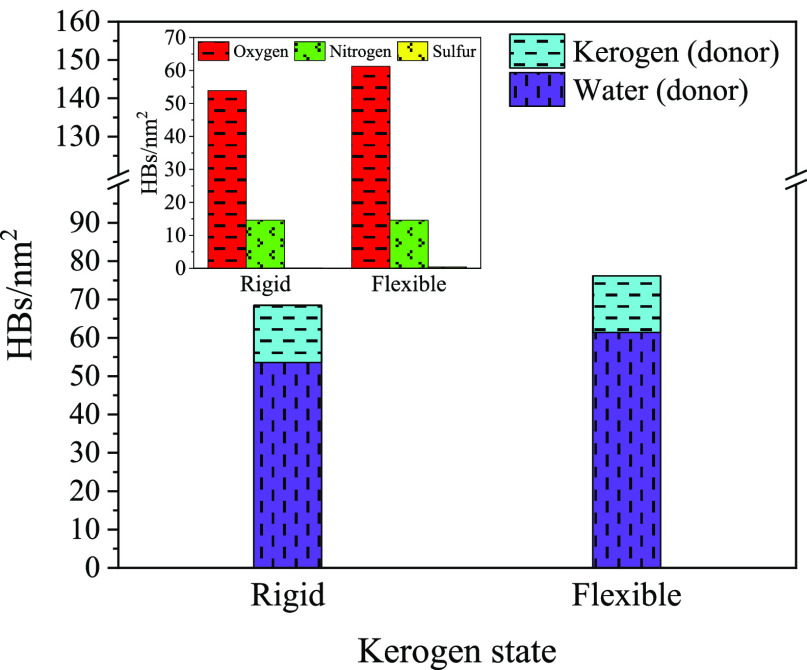
Hydrogen bonds between water and the rigid and flexible kerogen surfaces.

So far, our exploration of the reasons behind the higher hydrophilicity of the flexible kerogen surface has primarily focused on its larger effective surface area while not considering its intricate heterogeneous structure. Although kerogen is predominantly composed of hydrocarbons, leading to primarily dispersion interactions, the presence of heteroatoms (O, N, and S) in kerogen introduces hydrogen bonding alongside dispersion interactions (Figure 8). Understanding these hydrogen bonds (HBs) can provide additional insight into the distinct behaviors exhibited by the two states of kerogen upon exposure to water molecules.

Employing the standard Luzar–Chandler criterion,^65^ we define a HB when the distance between the donor and the acceptor is below 0.35 nm and the angle of the hydrogen-donor–acceptor is less than 30°. The number of HBs between water and the two different states of kerogen, both rigid and flexible, is presented in Figure 8. Overall, the number of HBs on the flexible kerogen surface exceeds those on the rigid one by a factor of *r*_HB_ = 1.11. While this rise in HBs aligns with the larger effective area of the flexible kerogen, the growth is somewhat less than anticipated based on a straightforward enlargement of the surface area. An intuitive proportionality relationship between HBs and surface area would suggest *r*_HB_ = *r*. Contrary to this expectation, the predominant factor behind the enlarged surface area seems to be the exposure of the hydrocarbon fractions of kerogen, which do not participate in HB formation.

Water molecules exhibit the highest affinity toward oxygen atoms within the kerogen structure, followed by nitrogen atoms. A closer look at the inset in Figure 8 reveals that the number of HBs between the water and sulfur atoms of kerogen is exceedingly low. These results are in agreement with a recent study,^14^ where a major amount of water molecules formed HBs with oxygen atoms in kerogen, followed by nitrogen and sulfur atoms. It is important to note that this result cannot be attributed only to the higher abundance of oxygen atoms in the molecular structure of kerogen compared with the other two heteroatoms. The number ratio of the O:N:S atoms is 13:5:2, corresponding to 1:0.38:0.15, which differs from the ratio of hydrogen bonding with these atom types, being 1:0.27:0.00 for the rigid and 1:0.24:0.00 for the flexible surface. Another factor to consider is that oxygen is more electronegative than nitrogen and sulfur, thus forming HBs with water molecules more readily. Hu et al.^2^ also indicated that the presence of oxygen atoms in the form of carbonyl functional groups on the graphene surface can alter the wettability from hydrophobic to hydrophilic, showing oxygen atoms’ importance in wetting. As expected, the flexible surface features more HBs formed by oxygen than the rigid one. What is interesting, however, is that the number of HBs formed by nitrogen atoms is identical for both surfaces. This observation highlights a nontrivial relationship among surface flexibility, effective surface area, and HB propensity.

We also decompose HBs into acceptor–donor roles. This involves assessing two scenarios: in the first, water molecules act as donors and heteroatoms of kerogen as acceptors; in the second, the roles are reversed, with heteroatoms of the kerogen surface being donors and water molecules acting as acceptors (see Figure 8). The results reveal that in the vast majority of HBs, water molecules play the role of the donor. Only 25% of electronegative heteroatoms in kerogen have a covalently bonded hydrogen atom and can act as the donor. This limited availability of donor sites among the heteroatoms in kerogen suggests that their contribution to hydrogen bonding interactions is relatively constrained compared to water molecules, which can act as both the donor and acceptor. It is further interesting that while the HBs in which kerogen serves as the acceptor increase by a factor of 1.15 as we transition from the rigid to flexible surface, those HBs in which kerogen acts as a donor remain unchanged. Building upon the prior breakdown of HBs according to the participating atoms, it becomes clear that the increased flexibility in kerogen primarily favors the formation of HBs with its oxygen atoms serving as acceptors.

## 4 Conclusions

This study evaluated the effect of droplet size and morphology—including spherical and cylindrical shapes—on the contact angle of flexible and rigid kerogen surfaces. We found a direct relationship between the droplet radius and its contact angle for both morphologies, which can be attributed to the complex chemical nature of kerogen, its heterogeneity, and roughness. Although it is organic matter, kerogen exhibits a hydrophilic character, with a contact angle ranging between around 64 and 76°, owing to heteroatoms in its molecular structure that are capable of forming hydrogen bonds with water. On rigid kerogen surfaces, the contact angle is greatly influenced by droplet size. In contrast, on flexible surfaces, the contact angle shows minimal variation with the droplet size. Our observations regarding surface roughness are supported by both the Wenzel model and direct surface area measurements.

At a critical contact angle of approximately 65–70°,^66,67^ the surface transitions from being underoil hydrophilic to being underoil hydrophobic. In other words, water competes with other hydrophobic fluids, including hydrocarbons and CO_2_, to wet the surface. The hydrophobic or hydrophilic nature of the surface is determined by whether its contact angle surpasses or falls below the critical threshold. With a contact angle ranging between 64 and 76°, type II-C kerogen is positioned precisely at this transition point. Consequently, minor alterations in its structural properties and surface roughness can result in markedly distinct behaviors regarding the preference between water and other fluids.

In the realm of GCS, the efficacy of the process is enhanced by a hydrophilic surface, which effectively hinders the migration of CO_2_ toward the surface. Conversely, when extracting hydrocarbons from shale reservoirs, the presence of hydrophilic surface rock proves unfavorable due to its inherent tendency to obstruct gas flow within porous media. Furthermore, this hindrance can be exacerbated during the hydraulic fracturing process, as the injection of additional water into the reservoir further impedes efficient gas production.

## Supporting Information Available

Molecular configuration of kerogen and additional information on the composition and structural parameters of type II-C kerogen; density plot of the equilibrated spherical water droplet on the rigid/flexible kerogen surface (PDF)
