# Supplementary material for: Wetting Behavior of Kerogen Surfaces: Insights from Molecular Dynamics
Source: Langmuir. 2024 Mar 7;40(11):5715–24. doi: 10.1021/acs.langmuir.3c03367 (PMC10956498; doi:10.1021/acs.langmuir.3c03367)
Supplement: Supplementary file 1 — la3c03367_si_001.pdf [file la3c03367_si_001.pdf]

Supporting Information

Wetting Behavior of Kerogen Surfaces: Insights from Molecular Dynamics

Neda Sanchouli [a,1], Saeed Babaei [b,1], Matej Kanduč [c], Fatemeh Molaei [d,e], Mehdi Ostadhassan [f*]*

[a] *Department of Petroleum Engineering, Shahid Bahonar University of Kerman, Kerman 7616914111, Iran*

[b] *Civil Engineering Faculty, K. N. Toosi University of Technology, Tehran 1969764499, Iran*

[c] *Department of Theoretical Physics, Jožef Stefan Institute, Jamova 39, Ljubljana, 1000, Slovenia*

[d] *Department of Mining and Geological Engineering, The University of Arizona, Tucson, AZ 85721, United States*

[e] *Stantec consulting company, USA*

[f] *Institute of Geosciences, Marine and Land Geomechanics and Geotectonics, Christian-Albrechts Universität, Kiel 24118, Germany*

Corresponding authors' emails: (M. K.) matej.kanduc@ijs.si; (M. O.) mehdi.ostadhassan@ifg.uni-kiel.de

[1] These authors contributed equally.

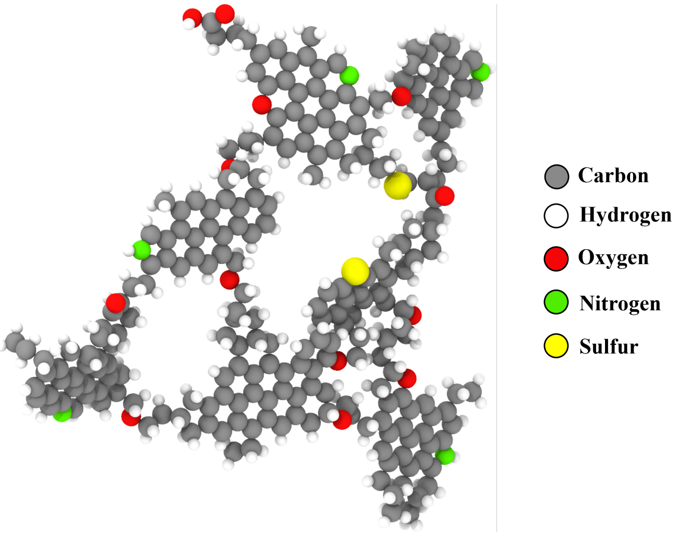

Figure S1. Molecular model unit of type II-C kerogen[1].

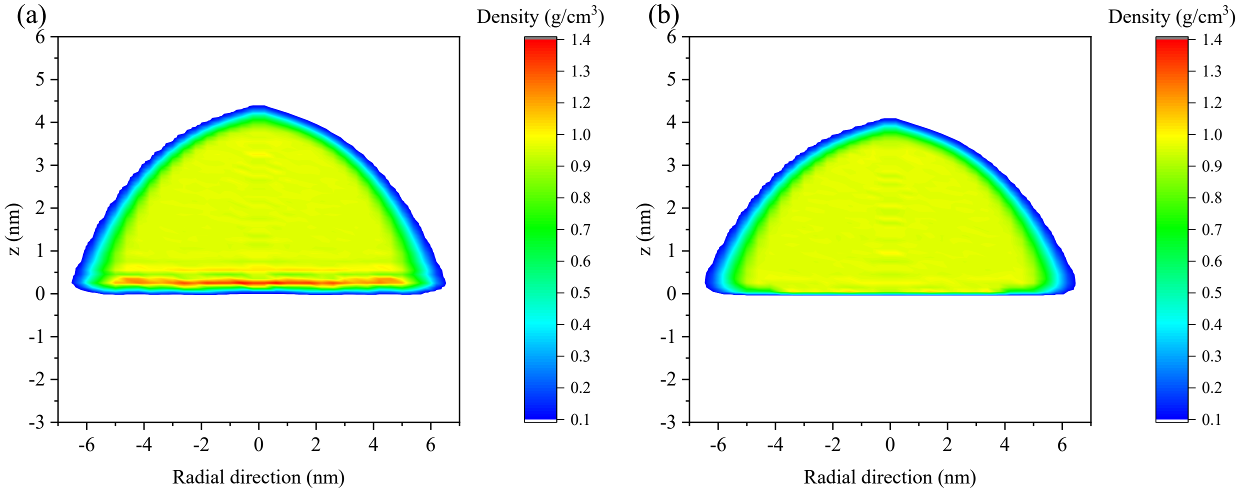

Figure S2. Density plot of an equilibrated spherical water droplet on a) the rigid and b) flexible kerogen surface.

Table S1. Composition and structural parameters of the kerogen model unit II-C[1].

Property	Parameter	Analytical data	Model unit
Composition	H/C	0.89	0.0905
	O/C	0.05	0.054
	N/C	0.021	0.021
	S/C	0.006	0.008
C group	Aromatic carbon from XPS (a) or NMR (b) (%)	54 (a), 54 (b)	58.7
	Avg. number of C atoms per aromatic cluster	19	20.3
	Fraction of aromatic carbon with attachments (sp3, C, N, S, O)	0.30	0.28
	Protonated aromatic carbons (per 100 C)	17	14
O group	Number of O in C-O per 100 C	3.5 (a), 5 (b)	3.7
	Number of O in carboxylic groups (-COOH) per 100 C	0.7	0.83
	Number of O in carbonyl groups (>C=O) per 100 C	0.8	0.83
N group	Pyrrolic (mol % of N)	65	60
	Pyridinic (mol % of N)	18	40
	Quaternary (mol % of N)	17	0
	Amino (mol % of N)	0	0
S group	Aromatic S (% of organic S)	54	50
	Aliphatic S (sulfides and thiols) (% of organic S)	46	50

References:

(1) Ungerer, P.; Collell, J.; Yiannourakou, M. Molecular Modeling of the Volumetric and Thermodynamic Properties of Kerogen: Influence of Organic Type and Maturity. *Energy & Fuels* **2015**, *29* (1), 91–105. https://doi.org/10.1021/ef502154k.
